# Canada’s changing climate: what does it mean for digestive health?

**DOI:** 10.1093/jcag/gwae052

**Published:** 2024-12-21

**Authors:** Desmond Leddin, Paul Sinclair, Harminder Singh, Rachael Sherman, Kelsey Cheyne

**Affiliations:** Division of Digestive Care and Endoscopy, Department of Medicine Dalhousie University, Victoria General Hospital, Halifax, NS B3H2Y9, Canada; INSINC Consulting Inc., Guelph, ON N1G 3G3, Canada; Section of Gastroenterology, Department of Internal Medicine, Rady Faculty of Health Sciences, Max Rady College of Medicine, University of Manitoba, Winnipeg, MB R3E 3P4, Canada; Section of Gastroenterology, Department of Biochemistry and Medical Genetics, Rady Faculty of Health Sciences, Max Rady College of Medicine, University of Manitoba, Winnipeg, MB R3E 3P4, Canada; Section of Gastroenterology, Department of Community Health Sciences, Rady Faculty of Health Sciences, Max Rady College of Medicine, University of Manitoba, Winnipeg, MB R3E 3P4, Canada; The Estée Lauder Companies, New York, NY 10153, United States; Canadian Digestive Health Foundation, 1540 Cornwall Road #224, Oakville, ON L6J 7W5, Canada

Canadians live in one of the areas with the most rapid pace of climate change. Canada is warming at twice the global average.^1^ While this has some advantages, such as a longer growing season, this change represents a major public health threat.^2^

Global warming, driven primarily by the burning of fossil fuels, is strongly correlated with pollution of water, air, and soil. Warming and pollution can adversely affect digestive health in several ways (Figure 1). Increasing temperatures make weather events such as storms, rainfall, and dry periods more extreme, and increase the likelihood of forest fires.^3^

**Figure 1.**
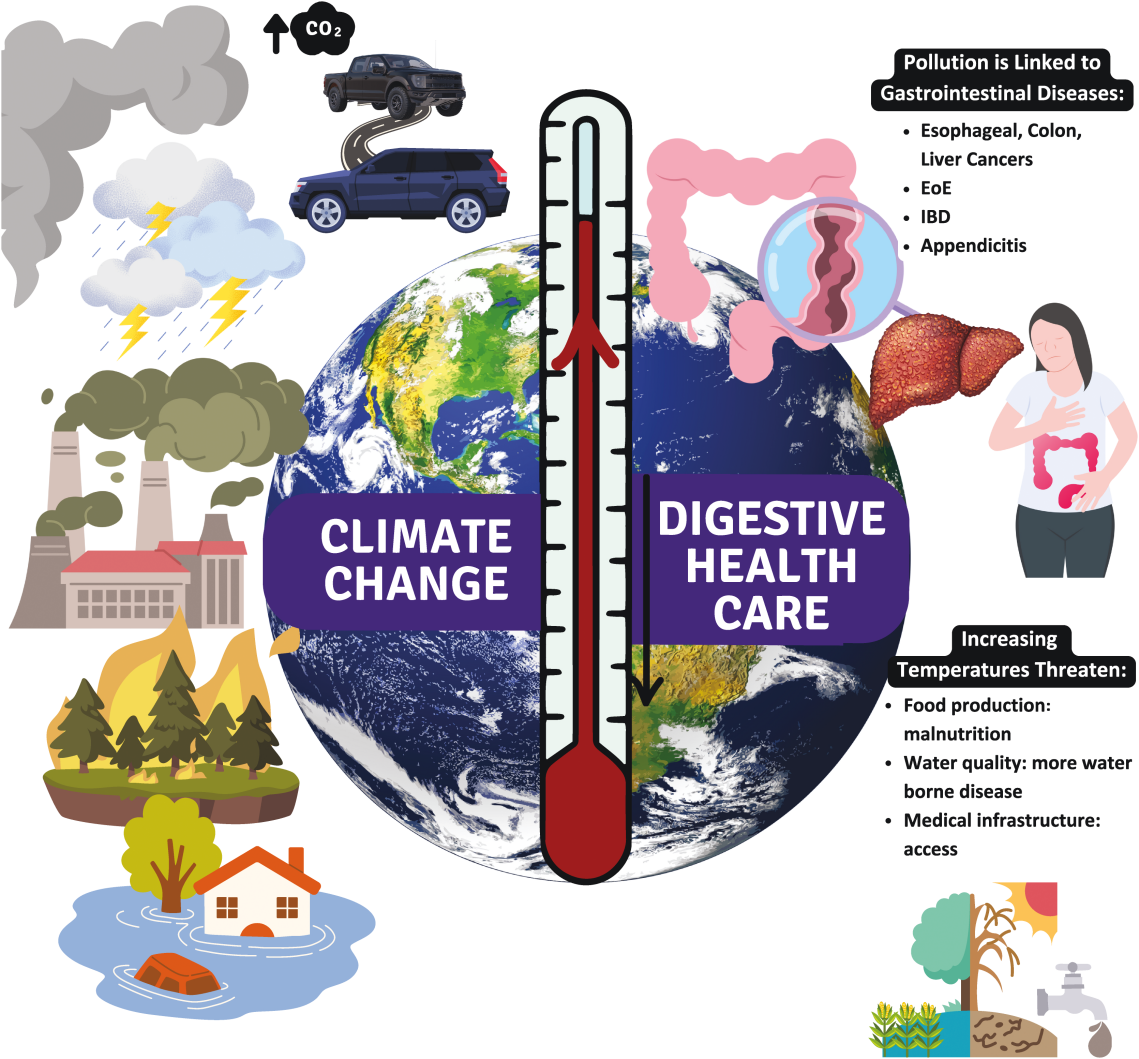
The relationship between climate change and digestive health.

These changes are already threatening water quality, food production, medical infrastructure, and supply chains. There is growing evidence for a connection between pollution and a variety of digestive illnesses including esophageal, colon, and liver cancers, eosinophilic esophagitis, appendicitis, and inflammatory bowel disease.^3^

Current digestive health delivery contributes to the problem because, as a high-volume diagnostic and therapeutic specialty, we generate a considerable amount of waste, pollution, and greenhouse gases.^4,5^ Endoscopy is the third largest generator of waste in hospital departments.^4^ Much of the waste is incinerated, adding to the pollution of the atmosphere.^6^

The Canadian Association of Gastroenterology (CAG) recognizes the need to minimize the environmental harm of practice and is releasing both its sustainability plan, and the methodology used to develop it.^7^ The plan will form the basis of CAG’s response to climate change and will help ensure that we deliver high-quality care while minimizing environmental harm.

## Supplementary Material

gwae052_suppl_Supplementary_Material

## Data Availability

No new data were generated or analysed in support of this manuscript.
